# Colistin for Treatment of Multidrug Resistant Central Nervous System Infection: A Triple Route Therapy!

**DOI:** 10.5005/jp-journals-10071-23183

**Published:** 2019-06

**Authors:** Ratnesh Kumar Shukla, Indu Kapoor, Charu Mahajan, Hemanshu Prabhakar

**Affiliations:** 1-4 Department of Neuroanesthesiology and Critical Care, All India Institute of Medical Sciences, New Delhi, India

## Abstract

**How to cite this article:** Shukla RK, Kapoor I, Mahajan C, Prabhakar H. Colistin for Treatment of Multidrug Resistant Central Nervous System Infection: A Triple Route Therapy!. Indian J Crit Care Med 2019;23(6):287.

Central nervous system (CNS) infection following neurosurgical procedures is life-threatening complication. The commonly detected pathogen is Staphylococci, but Gram-negative bacteria are also responsible for CNS infections.^1,2^ *Acinetobacter baumannii* (AB) is a common Gram-negative bacterium in the neurosurgical setting, followed by *Klebsiella pneumonia*.^3^ Colistin has been recommended for the treatment of infections by these multidrug resistant (MDR) Gram-negative rods.

A written informed consent was taken for publication from relatives as patient was on ventilatory support. We report a case of 65-year-old male diagnosed with subarachnoid hemorrhage (SAH) following a rupture of cerebral aneurysm who needed extra ventricular drain (EVD) and later developed ventriculitis during his intensive care unit (ICU) stay. His cerebrospinal fluid (CSF) examination revealed pleocytosis (TLC:140) and low protein (10 mg/dL) with normal sugar levels (60 mg/dL). Cerebrospinal fluid culture was suggestive of growth of AB sensitive to colistin. Frank pus drained from the EVD. A provisional diagnosis of ventriculitis/meningitis was made. His fever persisted and pus drainage continued even after starting the intravenous (IV) colistin 4.5 million units twice a day. Subsequently, it was decided to administer intrathecal (IT) colistin in the dose of 125000 units (U) through lumbar puncture. Extra ventricular drain was replaced by Omaya on day 16th and intra ventricular (IVT) colistin 125000 U twice a day was given along with IV colistin. He responded well to the treatment and remained afebrile during rest of his days in ICU.

*Acinetobacter baumannii* holds an important role in postoperative meningitis, and it can lead to mortality up to 15–71%. The incidence of EVD-associated infection ranges from 1 to 18%. Nowadays, IVT and IT colistin represents the last resort treatment of MDR Gram-negative rods ventriculitis/meningitis, offering a unique and successful mode of therapy. It has been observed that the combined IVT/IV administration of colistin results in higher CSF levels of drugs as compared to IV administration alone.^4^ In another study where IVT and IT colistin were compared, the rate of successful outcome was found to be 89%.^5^ We used all three routes of colistin administration in single patient. We would like to suggest that a combined triple route of IVT/IT and IV colistin in the management of severe CNS infections would possibly be more successful than a dual or single route specifically in patients with device related infection.
